# A new two-hit animal model for schizophrenia research: Consequences on social behavior

**DOI:** 10.1016/j.ibneur.2025.05.012

**Published:** 2025-05-28

**Authors:** Kristina Hakenova, Anna Mikulecka, Kristina Holubova, Marketa Chvojkova, Romana Slamberova, Jana Jurcovicova, Barbora Cechova, Silvester Ponist, Jiri Horacek, Karel Vales

**Affiliations:** a National Institute of Mental Health, Topolova 748, Klecany 250 67, Czech Republic; b Charles University, Third Faculty of Medicine, Ruska 87, Prague 100 00, Czech Republic; c Institute of Physiology CAS, Videnska 1083, Prague 142 20, Czech Republic; d Charles University, Third Faculty of Medicine, Department of Physiology, Ke Karlovu 4, Prague 120 00, Czech Republic; e Centre of Experimental Medicine SAS, Institute of Experimental Pharmacology & Toxicology, Dúbravská cesta 9, Bratislava 841 04, Slovak Republic

**Keywords:** Schizophrenia, Social behavior, negative symptom, Animal model, Two-hit model, Oxytocin

## Abstract

Schizophrenia, a profoundly impactful neuropsychiatric disorder, has been the subject of extensive research using animal models. However, certain important aspects remain understudied, including assumed long-term consequences of psychotic episodes on negative symptoms development and progression. Addressing these limitations, we proposed a novel animal model in male rats based on early postnatal immune activation triggered by lipopolysaccharide (LPS), serving as the predisposing factor (1st hit). As the 2nd hit, representing psychotic-like episodes, we implemented a multi-episodic co-treatment with dizocilpine (MK-801) and amphetamine (AMP), spanning multiple developmental periods. The animals were tested in two social behavioral assays in adolescence and adulthood to investigate whether a social deficit would arise. In addition, we evaluated the level of oxytocin (OT), a neuropeptide relevant to social behavior, in selected brain regions. In the social interaction test, when animals could freely interact in the open field and express their social behavioral profile entirely, social behavior decreased in adolescent experimental animals. In the social approach test in the Y maze, all animals, irrespective of treatment, preferred conspecific over an indifferent object and novel rat over a familiar rat. Further, the results revealed that the OT content in the hypothalamus increased with age. In the proposed model, social interaction in the open field was decreased in adolescent but not in adult rats, indicating that the pharmacological manipulations caused only transient age-dependent changes. The study was thus in certain aspects successful in creating a novel approach to model social deficit potentially relevant to schizophrenia; other findings require further investigation.

## Highlights

• A novel 2-hit developmental rat model for schizophrenia research.
• Postnatal immune activation and subsequent repeated psychotic-like episodes.
• Focus on the social deficit as one of the negative schizophrenia symptoms.
• The model led to a social deficit in adolescent, but not adult rats.
• Hypothalamic oxytocin level evaluation included.

## Graphical Abstract

**Figure:**
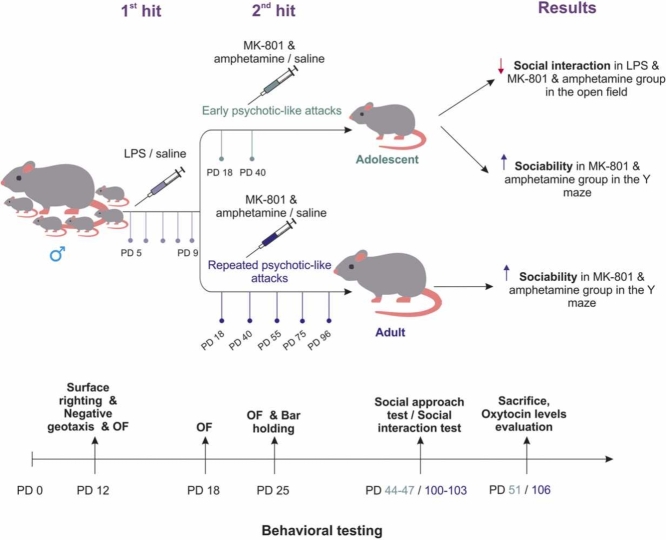

## 1 Introduction

Schizophrenia is a devastating neuropsychiatric disorder affecting 1 % of the global population, manifested by positive symptoms (hallucinations, delusions, disorganization), negative symptoms (social withdrawal, anhedonia, poverty of thought), and cognitive dysfunction (Brunet-Gouet and Decety, 2006, CSA Study Group, 2020, Owen et al., 2016). Between 1990 and 2019, the number of people with schizophrenia increased from 14.2 to 23.6 million (Solmi et al., 2023). While the complete pathophysiology of schizophrenia remains unclear, its neurodevelopmental nature has been firmly established. Both genetic (Gejman et al., 2010) and environmental factors (Lewis and Levitt, 2002, Stilo and Murray, 2019) during development can lead to the emergence of schizophrenia.

The two-hit hypothesis suggests that genetic susceptibility or environmental insults in the early developmental period serve as the first hit increasing vulnerability to the subsequent environmental factors (Bayer et al., 1999, Maynard et al., 2001), representing the second hit. During neurodevelopment or early childhood, infection can act as the first hit (Brown, 2011, Feigenson et al., 2014, Giovanoli et al., 2013). This immune activation may result in neurodevelopmental “priming,” rendering an individual more vulnerable to a second hit later in life (Galic et al., 2008). Environmental factors, representing 2nd hit in the following ontogenetic periods, may include infections (Brown, 2011), social adversity during childhood (Wicks et al., 2005), stress (Corcoran et al., 2003), and drug abuse (Hambrecht and Häfner, 1996). Both hits (biologically significant events) are necessary for disease onset.

According to the “neurotoxicity” hypothesis of schizophrenia, untreated psychotic episodes may be biologically toxic to the brain, potentially resulting in lasting functional consequences (Wyatt, 1991). Schizophrenia typically manifests as a multi-episodic disorder involving repeated psychotic attacks and phases of remission dominated by negative and cognitive symptoms (Bayer et al., 1999). Research results suggest that each untreated psychotic relapse may lead to progressive brain damage and contribute to the development of negative symptoms such as the social deficit, persistent into the non-psychotic phase (Maynard et al., 2001, Shepherd et al., 1989, Slováková et al., 2024, Wiersma et al., 1998, Yu et al., 2023). Negative symptoms are highly clinically relevant, as they are strongly associated with functional impairment and do not respond well to the treatment by current medications (Owen et al., 2016). Current schizophrenia research hence necessitates tools to investigate the mechanisms underlying the impact of psychotic relapses on the disease progression and explore therapeutic approaches to prevent it. Although there are many animal models of schizophrenia (Jones et al., 2011, Winship et al., 2019), in our opinion they do not focus much on these aspects.

Therefore, the primary objective of our study was to establish a two-hit animal model potentially relevant for schizophrenia, focusing on the delayed effects of a predisposing factor combined with later psychotic-like episodes on social behavior. Our research was based on the presumption that prenatal exposition to infection may represent a risk factor for schizophrenia (Brown, 2011) and that the severity of the negative symptoms may be enhanced by previous psychotic episodes (Slováková et al., 2024). We aimed to model this situation in male rats and investigate whether it would result in a social deficit. If so, the model may be helpful in schizophrenia research and it may support the neurotoxicity hypothesis.

Early-life immune activation by lipopolysaccharide (LPS) represented the first hit. LPS, an endotoxin routinely used in schizophrenia modeling (Harvey and Boksa, 2012, Kubesova et al., 2015), was administered on postnatal days (PD) 5–9. This age was selected according to our previous studies (Kubesova et al., 2015, Vojtechova et al., 2018) and is estimated to correspond to the last trimester of pregnancy and/or the perinatal period in humans (Clancy et al., 2007, McCutcheon and Marinelli, 2009, Semple et al., 2013). LPS administration in this period was proven to induce neurotransmitter system alterations in adulthood potentially relevant to schizophrenia, including excitatory-inhibitory imbalance, elevated dopamine levels and upregulated kynurenine pathway (Kubesova et al., 2015). This first hit aims to model a maternal infection during pregnancy as a predisposing factor for schizophrenia development.

For the 2nd hit, to induce psychotic-like phenotype, we used repeated dizocilpine (MK-801) and amphetamine (AMP) co-applications covering a broad range of the lifespan (PD 18, 40, 55, 75, and 96) covering the age of juvenility (PD18), adolescence (PD40), sexual maturity (PD55, PD75), and early adulthood (PD96). Instead of studying the acute behavioral effects of MK-801&AMP, we focused on the delayed effects of these manipulations.

There is converging evidence that MK-801 and AMP can mimic acute psychotic symptoms in humans and laboratory animals (Bubeníková-Valešová et al., 2008, Jones et al., 2011, Kantrowitz and Javitt, 2012, Lapish et al., 2014). MK-801 and AMP can to some extent replicate the glutamatergic hypofunction and the dysregulation of the dopaminergic system, respectively—two mechanisms supposed to be involved in the disease’s pathophysiology. Both drugs are regularly used in preclinical research for schizophrenia modeling (Bubeníková-Valešová et al., 2008, Jones et al., 2011) and we assumed that their co-application could replicate psychotic-like attacks more accurately. Specifically, MK-801 preferentially inhibits N-methyl-D-aspartate (NMDA) glutamate receptors located on inhibitory interneurons, leading to disinhibition of pyramidal neurons in the prefrontal cortex and resulting in an excitatory-inhibitory imbalance (Homayoun and Moghaddam, 2007). This imbalance in the prefrontal cortex may, in turn, contribute to increased dopamine release in the striatum (Miller and Abercrombie, 1996). We aimed to augment this effect by application of AMP (Miller and Abercrombie, 1996). By co-administering MK-801 and AMP during critical periods of brain development, we aimed to amplify the effects of prior LPS exposure by further enhancing excitatory-inhibitory imbalance in the PFC. The findings of Basta-Kaim et al., showing that previous early-life LPS administration may enhance the acute psychotomimetic effects of MK-801 (Basta-Kaim et al., 2011), seem consistent with this notion. We hypothesized that the pharmacological interventions (hits) received during development and early adulthood may result in a more profound schizophrenia-like behavioral phenotype (social deficit) in later behavioral testing.

In the present study, our primary focus was on assessing social behavior functioning, as its impairment represents one of the core negative symptoms of schizophrenia (Gururajan et al., 2010, Potasiewicz et al., 2020, Rich and Caldwell, 2015, Wilson and Koenig, 2014). The main aim of the study was to assess social behavior in two assays at two crucial developmental periods: adolescence (PD44) and adulthood (PD100). Experiments were performed 4–7 days after the last MK-801&AMP administration. Moreover, since potential impairments of sensorimotor function may confound behavior results (Crawley, 2008), the animals postnatally underwent a comprehensive battery of sensorimotor tests. Additionally, we evaluated the level of oxytocin (OT), a neuropeptide related to social behavior and schizophrenia (Feifel et al., 2016, Iovino et al., 2018, Lee et al., 2009, Rich and Caldwell, 2015), in selected brain structures. We focused on the hypothalamus as the site of OT production, and the striatum and prefrontal cortex, which are believed to play crucial roles in mediating OT's effects on negative symptoms and social cognition in schizophrenia (Feifel et al., 2016).

## 2 Materials and methods

### 2.1 Animals

Twelve litters of Wistar rats (Velaz Ltd., Czech Republic), each comprising 10 male pups, were used in the experiments. The animals were housed in the National Institute of Mental Health facility, Czech Republic, in standard transparent cages in a temperature-controlled room (23 ± 1 °C) with a 12-h light/dark cycle (light on at 6:00 h) and *ad libitum* access to food and water. The day of birth was defined as PD0. At PD5, littermates were pseudo-randomly and evenly distributed by treatment (i.e., half of each litter received LPS, and the other half received saline). At PD27, weaned rats were housed in groups of 2–3. The experiments were performed during the light phase of the day. The experiments were conducted following the guidelines of the European Union directive 2010/63/EU and Act No 246/1992 Coll., on the protection of animals against cruelty and were approved by the Animal Care and Use Committee of the National Institute of Mental Health (reference number MZDR 14764/2019–4/OVZ).

### 2.2 Drugs

Lipopolysaccharide (LPS) E. coli, serotype 026:B6, (+)-MK-801 hydrogen maleate (MK-801), and D-amphetamine sulfate (AMP; all from Sigma Aldrich, Czech Republic) were dissolved in saline and administered intraperitoneally (i.p.). LPS (or saline) was applied at 2 mg/kg/day (injection volume 10 mL/kg at PD5–6 and 5 mL/kg at PD7–9). MK-801 at 0.3 mg/kg was co-administered with AMP at 2 mg/kg (the volume of each injection was 2.5 mL/kg at PD18 and 1 mL/kg at PD40 and older; injections were administered one right after the other). Control animals received corresponding volumes of saline. (MK-801 and AMP doses are expressed as the salt form of the drugs.)

When selecting the doses, the literature was consulted (Andiné et al., 1999, Gururajan et al., 2010, Vojtechova et al., 2018). In our opinion, the selected doses of MK-801 and AMP are considered medium-to-high in the context of rodent pharmacological research (Andiné et al., 1999, Danysz et al., 1994, Gururajan et al., 2010, Wegener et al., 2011) and are known to induce hyperlocomotion and some other psychotomimetic manifestations in rats (Andiné et al., 1999, Danysz et al., 1994, McDougall et al., 2020, Vasilev et al., 2003, Wegener et al., 2011).

### 2.3 Group design and model induction

Animals were pseudo-randomly ascribed into treatment groups (1st and 2nd hit combinations). The 1st and 2nd hits were administered at defined PDs. Moreover, the groups were split into subgroups depending on the number of 2nd hit (MK-801&AMP, or saline) administrations and consequently the age at behavioral testing (adolescents vs. adults). Separate sets of animals were used for defined sets of experiments (Table 1).

**Table 1:** Experimental design.

	**Group name**	**Drug administration**			**Tested as**	**n**
		**1**^**st**^**hit**	**2**^**nd**^**hit**			
		**PD 5–9**	**PD 18, 40**	**PD 55, 75, 96**		
**Litters 1–9:**						
	saline	saline	saline	-	adolescents	9
	LPS	LPS	saline	-	adolescents	10
	MK−801&AMP	saline	MK−801&AMP	-	adolescents	9
	LPS + MK−801&AMP	LPS	MK−801&AMP	-	adolescents	10
	saline	saline	saline	saline	adults	10
	LPS	LPS	saline	saline	adults	10
	MK−801&AMP	saline	MK−801&AMP	MK−801&AMP	adults	10
	LPS + MK−801&AMP	LPS	MK−801&AMP	MK−801&AMP	adults	9
**Litters 10–12:**						
	saline	saline	saline	-	adolescents	8
	LPS + MK−801&AMP	LPS	MK−801&AMP	-	adolescents	8
	saline	saline	saline	saline	adults	11
	LPS + MK−801&AMP	LPS	MK−801&AMP	MK−801&AMP	adults	10

Note: n indicates the number of subjects per group. Litters 1–9 were used for the sensorimotor tests and the social approach test in the Y maze, and litters 10–12 were used for the open field test, the social interaction test, and the OT evaluation. 1st hit involved five injections of LPS or saline, 2nd hit involved two co-administrations of MK-801&AMP or saline in adolescent animals, or five co-administrations of MK-801&AMP or saline in adult animals (on defined PDs).

Following drug administration, the rats were promptly returned to their home cages. In the group receiving MK-801&AMP, a marked increase in locomotor activity was observed within 1–2 hours post-treatment (not quantified). This behavioral response aligns with previous findings associating hyperlocomotion with psychotomimetic states (Andiné et al., 1999, Bubeníková-Valešová et al., 2008, Jones et al., 2011, Manahan-Vaughan et al., 2008).

### 2.4 Behavioral testing

All behavioral testing was performed without acute drug administration on the testing day.

#### 2.4.1 Sensorimotor performance in immature rats

Immature animals were submitted to an established battery of tests selected for their developmental appropriateness (Mikulecká and Mareš, 2002) to evaluate whether LPS affects sensorimotor performance. The following tests were performed: surface righting at PD12; negative geotaxis and open field at PD12 and PD18; and bar holding and open field at PD25 (Mikulecká and Mareš, 2002). In addition, the rats were weighed between PD5–PD18 to evaluate whether LPS affected body growth. At PD12 and PD18 two groups of animals were tested (saline, n = 38, LPS, n = 39); at PD25 four groups of animals were tested (saline, n = 19; LPS, n = 20; MK-801&AMP, n = 19; LPS + MK-801&AMP, n = 19).

##### 2.4.1.1 Surface righting test

The rat was placed in the supine position on a flat surface. The latency to righting to the upright position was recorded for 60 s. The test was repeated three times, and the mean value was calculated.

##### 2.4.1.2 Negative geotaxis

The rat was placed on an inclined rough surface (30°) with its head pointing downwards. The latency of turning around (180° turn) with the head pointing upwards was measured. A maximum time limit of 90 s was imposed for each trial.

##### 2.4.1.3 Bar holding

A wooden round bar (65 cm long, 8 mm diameter) was suspended horizontally, 25 cm above a soft surface. The rat was held by the nape and allowed to grasp the bar with its forepaws. The latency of the fall was measured (maximum limit 120 s).

##### 2.4.1.4 Open field test in immature rats

The rat was allowed to move freely in a black plastic square arena (40 × 40 cm) for 10 min. Then, the distance traveled was analyzed using tracking software (EthoVision, Noldus Information Technology, Netherlands).

#### 2.4.2 Social approach test in the Y maze

The social approach test was performed at PD47 (adolescents), resp. PD103 (adults), i.e., 7 days after the last drug administration. The procedure tested sociability and social novelty preference. For the treatment groups studied and the number of animals per group see Table 1.

Many studies validated the procedure's utility for evaluating social behavior in rats and mice (Bitanihirwe et al., 2010, Mikulecka et al., 2019, Moy et al., 2004, van der Kooij and Sandi, 2012). Rats generally prefer to explore and interact with conspecifics rather than inanimate objects (Latané and Werner, 1971). The test was performed in a modified Y maze, consisting of three identical arms (10 × 50 cm each), radiating from a central triangle to form a “Y” shape with a 120° angle between arms. The arms were surrounded by 30 cm high walls (white plastic). Two arms were divided by a vertical metal grid. The grids were installed near the end of the arms, distant from the central triangle, creating 15.5 cm long enclosures at the ends of the arms, where the stimuli rats/object were placed (see later). The third arm served as a start zone. The experimental design was modified according to (Richetto et al., 2015, Weber-Stadlbauer et al., 2017). The experiment was performed for two consecutive days. On the first day, the rat was released in the start zone and was allowed to move freely in the maze for 10 min for habituation. On the second day, the test consisted of two trials. During the first trial, an unfamiliar male rat of the same strain and matching body weight (stimulus rat) was enclosed in one arm of the maze. An inanimate object (metal cylinder, 16 cm height) was placed in the other arm. The rat/object location was selected pseudo-randomly and counterbalanced between animals (i.e., approximately half of the animals in each treatment group were tested with the stimulus rat placed in the right arm, while the other half were tested with the stimulus rat placed in the left arm). The experimental rat was released in the start zone and allowed to explore the apparatus for 10 min (test for sociability). The second trial tested social recognition (social novelty preference) as immediate retention. The tested rat was removed from the maze and kept in a plastic box. The stimulus rat from the first trial always stayed in the same enclosure of the maze arm, becoming a familiar rat in the second trial. The object was removed from the second arm of the maze, and an unfamiliar male rat of the same strain and matching body weight was placed there. The experimental rat was then released in the start zone and allowed to explore the apparatus for 10 min.

The experiment was monitored by a camera mounted above the maze. Behavioral performance was analyzed by tracking software (EthoVision, Noldus Information Technology, Netherlands) using three body point detection (nose, center point, tail base). The parameter evaluated in the experimental animal was the cumulative duration of the investigation, defined as the animal’s nose being within a 3 cm interaction zone surrounding the grids enclosing either the object or stimulus rat. The percentage of time spent investigating the stimulus rat relative to the total time spent investigating both enclosures of the Y maze was calculated. This calculation was performed using the formula: (time_stim_/(time_stim_ + time_object or familiar rat_)) × 100. Time_stim_ stands for the duration of the investigation of stimulus represented by the rat in the sociability test and the unfamiliar rat in the social novelty preference test.

During the habituation phase, neither treatment group showed a significant side preference (time spent in the right/ in the left arm of the maze was calculated and compared to the value “1” by one-sample *t*-test or one-sample Wilcoxon test, yielding no significant differences; data not shown).

#### 2.4.3 Open field test and subsequent social interaction test in the open field

The social interaction test was performed at PD44 (adolescents), resp. PD100 (adults), i.e., 4 days after the last drug administration. For the treatment groups studied and the number of animals per group see Table 1. Before the social interaction test*,* the animals were habituated to the apparatus individually, enabling analysis of the locomotor activity and within-session habituation in the open field.

##### 2.4.3.1 Open field test

The animals were transferred to the experimental room 40 min before testing. The test was performed in an open field arena (80 × 80 × 40 cm, black plastic) 2 days before the social interaction test. The experimental animals were monitored individually for 10 min in the open field. Besides total traveled distance (cm), distance moved in 2-min intervals was analyzed to assess the within-session habituation (Mikulecka et al., 2019, Mikulecka et al., 2014) using tracking software (EthoVision, Noldus Information Technology, Netherlands). The open field test allowed the experimental animals to habituate to the arena where social interaction took place. The purpose was to familiarize rats with the arena to promote social interactions. Stimulus rats were familiarized with the arena for two consecutive days, in pairs with another partner taken from a different home cage, to get acquainted with the experience of encountering a novel conspecific in the arena.

##### 2.4.3.2 Social interaction test in the open field

The test has been widely used and extensively validated as a rat model of anxiety-like behavior (File and Seth, 2003, Lapiz-Bluhm et al., 2008, Šlamberová et al., 2015b). The rats were paired with a "stimulus" rat for age, gender, and body weight (within ± 10 g). Testing started by placing the experimental and stimulus rats at opposite corners of the open field arena, facing away from each other. A video camera recorded the 10-min test for offline analysis by two experimenters blinded to the treatment condition of the animals. The dependent measure was the time the experimental animal engaged in active social behavior with an unfamiliar "stimulus" rat (cumulative duration). The time spent in active social behavioral patterns initiated by the experimental animal was scored: nose contact (mutual sniffing), body contact (rats in direct contact, bodies touching each other), following (pursuing the stimulus rat), genital investigation (sniffing the anogenital area), and crawling (climbing over/under) (Mikulecka et al., 2014, Šlamberová et al., 2015a). Total social interaction time was calculated by summing the duration of these behaviors. Pearson correlation confirmed a good agreement between the observers in measuring social behavior (r = 0.974; p = 0.001).

### 2.5 Oxytocin (OT) level evaluation

At PD51 or PD106, the rats (adolescent groups: saline, LPS + MK-801& adult groups: saline, LPS + MK-801&AMP) were deeply anesthetized by an overdose of chloral hydrate anesthesia (40 mg/kg, *i.p.*). The brains were *in situ* transcardially perfused with 50 mL of physiological saline, removed, and the hypothalamus, the prefrontal cortex, and the striatum were dissected, snap-frozen in dry ice, and stored at –63 °C until extraction.

The brain structures were homogenized in ice-cold physiological saline (10 mg/100 µL) containing protease inhibitor cOmplete (Roche), 1 Tbt per 50 mL Ultra-Turrax homogenizer. The homogenates were sonicated using Qsonica Sonicator Q700 and centrifuged at 14000 g and 4 °C for 10 min. OT was extracted from the supernatant. Solid phase extraction was performed on C18Sep-Pak columns equilibrated with 3 mL acetonitrile, twice washed with 3 mL of 0.1% trifluoroacetic acid. The flow-through was discarded, and after the washing of the column, OT was eluted with 3 mL of 60% acetonitrile; the solvent was evaporated under nitrogen, and the dried samples were stored at −70 °C until assayed.

The quantitation of OT (Szeto et al., 2011) was performed using an Oxytocin ELISA kit from Enzo (cat. No. ADI-900–153A) according to the manufacturer's instructions. Just before the assay, the dried extracts were dissolved in the assay buffer as follows: hypothalamus in 230 µL, prefrontal cortex, and striatum in 110 µL. For analysis, 100 µL of samples was taken except for the hypothalamus when 23 µL was used. Within the assay coefficient of variance (CV) was 6 or 10 % and between assays CV was 14 %. Samples with OT levels below the detection limit were excluded from the analyses.

### 2.6 Data analysis

The data was analyzed using GraphPad Prism 8.0 software (San Diego, CA, USA). The results were expressed on graphs as mean + SEM. Differences were considered significant if *p* < 0.05. Statistical analyses used were *t*-test (surface righting), two-way ANOVA (open field at PD25, bar holding test at PD25, and social approach test in the Y maze with factors 1st and 2nd hit; open field test at adolescence/adulthood, social interaction and OT levels with factors age and treatment), and two-way ANOVA repeated measures (body weight, negative geotaxis and open field at PD12, PD18 with factors PD and 1st hit; within-session habituation with factors time interval and treatment). Statistical analysis was followed by Sidak's multiple comparison test when appropriate. When the assumption of normality was not met, the data were transformed using a logarithmic transformation. For better clarity, the original datasets are illustrated in the graphs.

## 3 Results

### 3.1 Body weight PD5–PD18

**Fig. 1:**
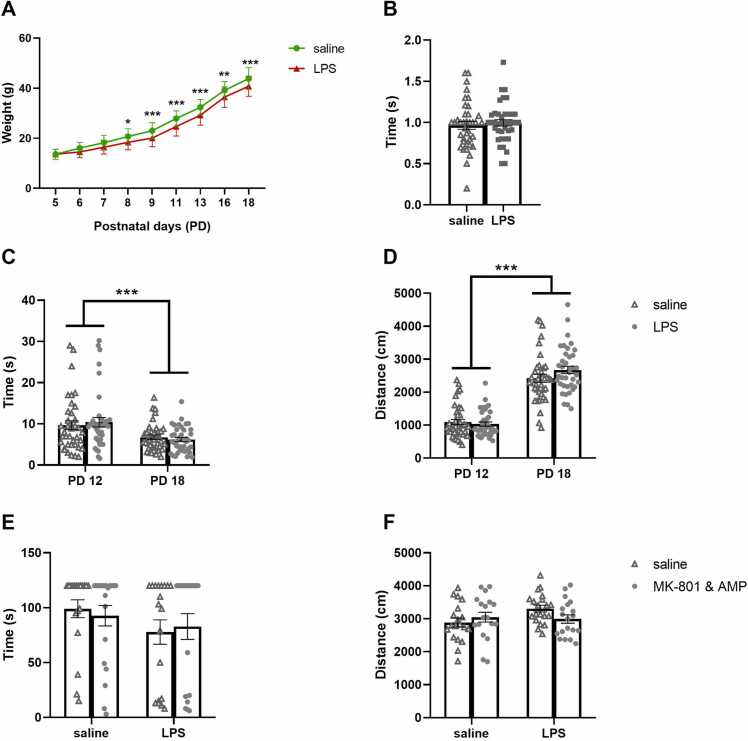
**Body weight and sensorimotor testing in immature rats.** Body weight at PD5– PD18 in pups treated with saline or LPS (A). Surface righting at PD12 (B). Negative geotaxis (C) and open field test (D) at PD12 and PD18. Bar holding (E) and open field test (F) at PD 25 (seven days after the first MK-801&AMP administration). LPS decreased body weight without affecting sensorimotor performance. Mean + SEM values, *p < 0.05, * *p < 0.01, * **p < 0.001.

LPS-treated animals had lower body weight than controls from PD8 until PD18, although the rate of weight gain was similar in both groups. Analysis showed a significant effect of interaction PD × 1st hit [F(8, 600)= 14.21, *p* < 0.0001], the effect of PD [F(8, 600)= 5356, *p* < 0.0001], and the effect of 1st hit [F(1, 75)= 11.13, *p* = 0.0013] (Fig. 1**A**). Post hoc test found a significant difference in weight between the groups at PD8 (*p* = 0.0182), PD9 (*p* = 0.0006), PD11 (*p* = 0.0001), PD13 (*p* = 0.0002), PD16 (*p* = 0.0028) and PD18 (*p* = 0.0003).

### 3.2 Sensorimotor performance in immature rats

#### 3.2.1 PD12, PD18

There was no significant effect of previous LPS treatment (PD5–9) on surface righting at PD12 (Fig. 1**B**), negative geotaxis at PD12 and PD18, or locomotion in the open field at PD12 and PD18. However, the sensorimotor performance was improved between PD12 and PD18 as a function of age, for negative geotaxis [F(1, 75)= 16.42, *p* = 0.0001] (Fig. 1**C**), and for open field [F(1, 74)= 399.8, *p* < 0.0001] (Fig. 1**D**).

#### 3.2.2 PD25

The pups were administered with the first MK-801&AMP injection at PD18 and subsequently tested in bar holding and open field one week later. There was no observed effect on bar holding (Fig. 1**E**) or open field activity (Fig. 1**F**) due to either LPS or MK-801&AMP.

### 3.3 Behavior in adolescent and adult rats

#### 3.3.1 Social approach test in the Y maze

**Fig. 2:**
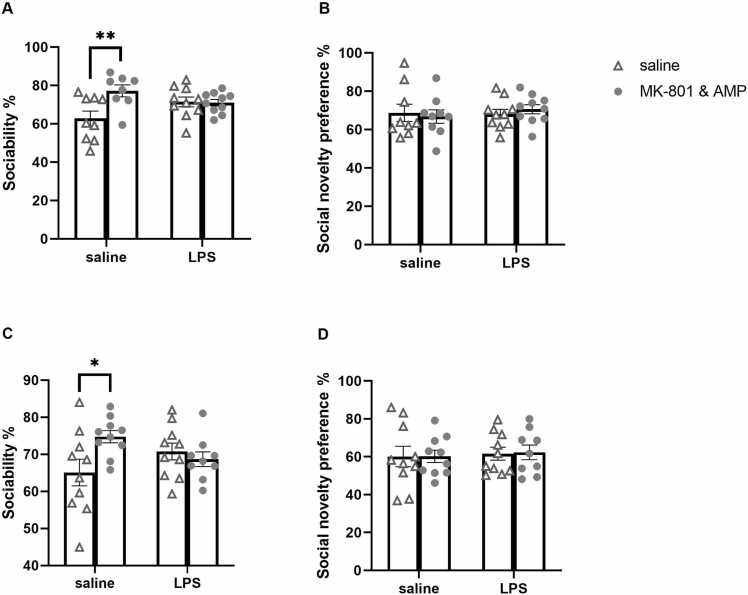
**Social approach test in the Y maze**. Effect of early-life LPS administration and/or subsequent MK-801&AMP administrations on sociability and social novelty preference in adolescent (A, B) and adult (C, D) rats. The percentage of time spent investigating the stimulus rat in the sociability test (or the novel stimulus rat in the social novelty preference test) relative to the total time spent investigating both enclosures of the Y maze is shown. Treatments failed to cause deficits in sociability and recognition memory in both age groups. Instead, MK-801&AMP increased sociability in saline-treated adolescents and adults. Mean + SEM values, *p < 0.05, * *p < 0.01.

In the sociability phase of the test, all the treatment groups spent > 50 % of the investigation time investigating the stimulus rat, indicating that they preferred the rat over the inanimate object. In the sociability analysis, two-way ANOVA revealed a significant effect of 2nd hit [F(1, 33)= 6.012, *p* = 0.0197] and interaction of 1st hit × 2nd hit [F(1, 33)= 6.720, *p* = 0.0141] on adolescent rats. Sidak's multiple comparisons test found a difference between saline and MK-801&AMP groups (*p* = 0.0033). Specifically, administration of MK-801&AMP to saline-treated adolescents increased the proportion of time spent investigating a conspecific (Fig. 2**A**). Similarly, in adult rats, a significant effect of the interaction of 1st hit × 2nd hit [F (1, 35)= 5.466, *p* = 0.0252] was detected by two-way ANOVA. Sidak's post hoc test showed a difference between saline and MK-801&AMP adult groups (*p* = 0.0182, Fig. 2**C**). On the other hand, the combination of the two hits or LPS alone had no negative impact on sociability.

In the social novelty preference test, all the groups preferred the novel rat over the familiar rat. Social novelty preference remained unaffected by treatment in both age groups, adolescents (Fig. 2**B**) and adults (Fig. 2**D**). Therefore, our animal model did not produce any negative schizophrenia symptoms in this experimental setup.

#### 3.3.2 Locomotor activity and within-session habituation in the open field

**Fig. 3:**
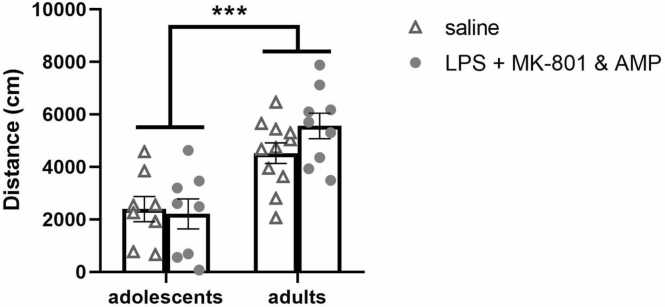
**Open field test.** Effect of early-life LPS administration and subsequent MK-801&AMP administrations on the distance traveled during the 10-min exposure to the open field. The adult rats walked significantly longer distances compared to adolescents. Treatment had no adverse effect on activity in the open field. Mean + SEM values, * **p < 0.001.

The adult animals traveled a longer distance than adolescents, irrespective of treatment. ANOVA revealed a significant effect of age [F(1,32)= 32.71, *p* < 0.001]. However, no significant effect of treatment was found (Fig. 3). Analysis of within-session habituation (distance moved in 2-min time intervals) revealed the effect of time interval [F(4, 56)= 6.69, p = 0.0002 and F(4, 72)= 16.49, *p* < 0.0001 for adolescent and adult groups, respectively], but no effect of treatment (data not shown). The analysis confirmed that with increasing time, the locomotion decreased, indicating within-session habituation in all the groups.

#### 3.3.3 Social interaction test in the open field

**Fig. 4:**
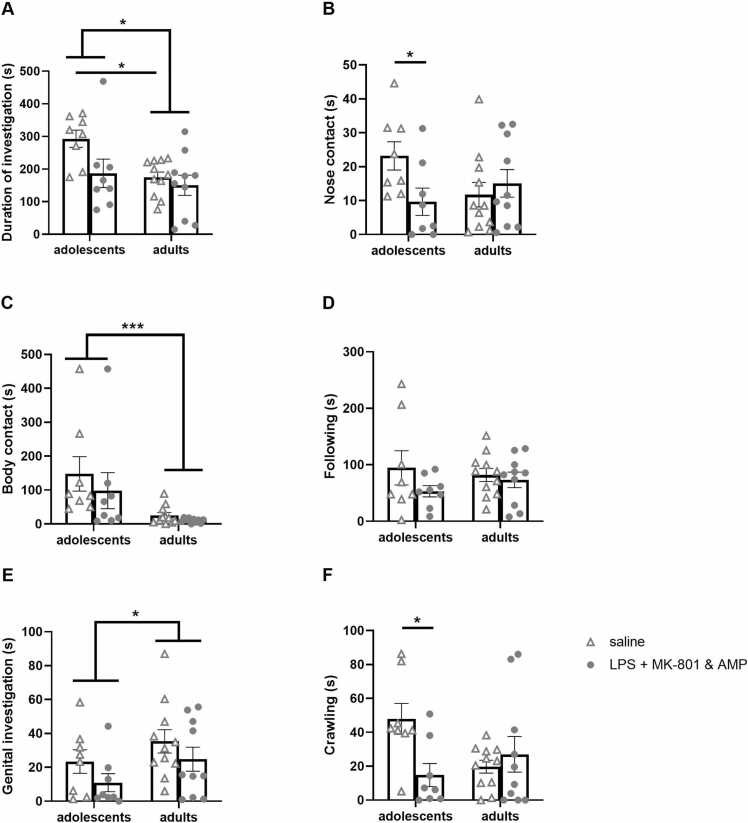
**Social interaction in the open field**. Effect of early-life LPS administration and subsequent MK-801&AMP administrations on the behavioral profile of adolescent and adult rats expressed during social interaction in the open field. Total time spent in social interactions with the “stimulus” rat (A). Duration of individual patterns of social behavior: nose contact (B), body contact (C), following (D), genital investigation (E), and crawling (F). In adolescents, LPS + MK-801&AMP decreased nose contact and crawling time. Mean + SEM values, *p < 0.05, * **p < 0.001.

The overall analysis of time spent in social interaction revealed a significant effect of treatment [F(1, 33)= 5.51, *p* = 0.0251], a significant effect of age [F(1, 33)= 6.04, *p* = 0.0194], and no significant effect of treatment × age interaction. As confirmed by subsequent post hoc analysis, the adolescent control animals spent more time interacting with conspecific than adult control animals (*p* = 0.0394). In adolescence, there was a tendency for decreased social behavior in the LPS + MK-801&AMP group compared to controls (*p* = 0.0685). In adulthood, the analysis did not reveal any difference between the control and experimental group (Fig. 4**A**).

The evaluation of individual patterns of social behavior revealed a significant effect of age × treatment interaction on nose contact time [F(1, 33)= 5.46, *p* = 0.0257]. Subsequent analysis showed that the adolescent control animals spent a longer time in nose contact than adolescent LPS + MK-801&AMP animals (*p* = 0.0283, Fig. 4**B**). The effect of age was detected in body contact [F(1, 33)= 20.33, *p* < 0.0001] (Fig. 4**C**) and genital investigation [F(1, 33)= 4.18, *p* = 0.0490] (Fig. 4**E**) analysis. However, while body contact time in adolescents was increased compared to adults, in the genital investigation, adolescents spent less time investigating the anogenital area of the conspecific than adults. Regarding the following of the intact rat by the experimental rat, none of the factors or their interaction had a significant effect on this parameter (Fig. 4**D**). Finally, a significant treatment effect on crawling time was revealed [F(1, 33)= 4.41, *p* = 0.0434] (Fig. 4**F**). The post hoc test found significantly shorter crawling time in the adolescent LPS + MK-801&AMP group than in the adolescent saline-treated group (*p* = 0.0203).

### 3.4 OT levels in the prefrontal cortex, striatum, and hypothalamus

**Fig. 5:**
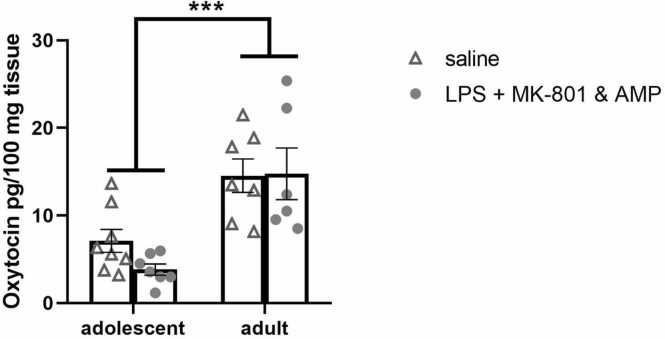
**Oxytocin (OT) levels**. Effect of early-life LPS administration and subsequent MK-801&AMP administrations on the levels of OT in the hypothalamus. OT levels differed significantly between adolescent and adult rats. Mean + SEM values, * **p < 0.001.

Since the analysis of the OT concentrations in the prefrontal cortex (**Suppl. 1 A**) and striatum (**Suppl. 1B**) did not reveal any differences between groups, these results are shown in the supplementary material only. The analysis of OT concentrations in the hypothalamus revealed the effect of age [F(1, 24)= 26.75, *p* < 0.0001], with adult animals displaying higher levels of OT (Fig. 5).

## 4 Discussion

Prenatal exposition to infection is considered a risk factor for schizophrenia (Brown, 2011). Besides, it has been suggested that schizophrenia progression and negative symptom development may be driven by pathophysiological processes induced by previous psychotic episodes experienced by the individual (Shepherd et al., 1989, Slováková et al., 2024, Wiersma et al., 1998). Understanding these processes may be critical for schizophrenia research and the development of proper treatment. Therefore, we proposed a novel two-hit animal model incorporating these manipulations and investigated their effects on social behavior.

The presented animal model is based on the exposure of animals to an early postnatal immune activation by LPS (1st hit; (Boksa, 2010; Harvey and Boksa, 2012; Kubesova et al., 2015; Meyer, 2013; Vojtechova et al., 2018)), serving as a predisposing factor, and subsequent exposure to repeated co-administrations of AMP and MK-801 (2nd hit), intended to model certain aspects of psychotic-like episodes (Bubeníková-Valešová et al., 2008, Jones et al., 2011). It was hypothesized that subjecting the animals to five AMP and MK-801 co-administrations during life would lead to a more profound schizophrenia-like phenotype in the delayed behavioral testing (performed 4–7 days after the last MK-801&AMP administration), i.e., a more profound social deficit.

Postnatal immune activation in animals treated with LPS was associated with lower body weight than controls from PD8 to PD18. However, this weight difference vanished in adolescence, suggesting that body weight reduction was not a persistent consequence. This observation aligns with studies showing that LPS administration at various times during the neonatal period has no long-term effects on body weight (Kubesova et al., 2015, Spencer et al., 2007). Although the LPS-treated pups showed lower body weight, this deficit did not significantly affect their sensorimotor performance. Moreover, the locomotion and adaptive response over time (within-session habituation) in the open field in adolescence and adulthood were also unaffected. These results suggest that sensorimotor performance did not interfere with social behavior in our experimental setup.

Schizophrenia is fundamentally a disorder of the social brain (Brunet-Gouet and Decety, 2006, Porcelli et al., 2019, Wilson and Koenig, 2014). Although it is impossible to model the full phenotypic spectrum of schizophrenia in animals, rats, being social animals with a rich repertoire of social behaviors, represent suitable candidates for modeling social behavior deficits. Social behavior in rats encompasses play behaviors, affiliative social behavior, preference, or recognition/discrimination, that can be successfully tested (Mikulecka et al., 2019, Mikulecka et al., 2014, Peters et al., 2017, Siviy and Panksepp, 2011, Yamamuro, 2006). Social behavior has natural rewarding properties in humans and animals (Ellenbroek and Youn, 2016, Krach et al., 2010, Manduca et al., 2021, Trezza et al., 2011). Therefore, social behavior in rats represents a relevant measure for investigating social dysfunction in schizophrenia (Gururajan et al., 2010, Potasiewicz et al., 2020, Wilson and Koenig, 2014). Considering the quantity of the experimental groups included, and for better comparability with existing models, only male rats were used in the current initial phase of model establishment. Nevertheless, we admit that the omission of females represents a limitation of our study.

In the present study, we characterized social performance at two ages in two tests. First, sociability and social novelty preference tests were performed using the social approach test in the Y maze. In this test, besides the groups receiving both hits (LPS + MK-801&AMP) and saline groups, groups receiving LPS only and MK-801&AMP only were included, to investigate possible interactions between the hits. Nevertheless, all the treatment groups of adolescent and adult rats spent less time investigating the indifferent object than investigating a conspecific in the sociability phase of the social approach test. Further, the rats preferred to investigate the novel rat over the familiar rat in the social novelty preference phase of the test. These results generally suggest an intact form of sociability and discrimination between conspecifics in adolescent and adult rats previously treated with LPS, MK-801&AMP, or LPS + MK-801&AMP. We did not prove a social deficit in any group. Instead, adolescent and adult rats treated with MK-801&AMP (without LPS) showed increased sociability. These results are rather unexpected, as MK-801 and AMP are usually used to model social withdrawal (Gururajan et al., 2010). Nevertheless, both drugs have also been found to increase social behavior in specific settings (Gururajan et al., 2010). However, it remains unclear to us, why the MK-801&AMP groups show increased sociability, while the behavior of the LPS + MK-801&AMP groups is unaffected.

Although negative in the context of the social approach, the absence of a social deficit in the LPS + MK-801&AMP groups does not necessarily invalidate the presented model. Schizophrenia's complexity warrants further characterization of the model in additional relevant behavioral tests, including cognitive assessments and exploration of interactions between the hits. This will be the subject of a separate upcoming study. In response to the negative findings from the social approach test in the Y maze, we incorporated the social interaction test in the open field with separate sets of animals, where the animals could freely interact and express their social and behavioral profile in a more natural environment, enabling a more detailed analysis. In this test, only the key groups of animals (saline; LPS + MK-801&AMP) were included. In addition, these experimental groups were used for the analysis of OT levels.

In the social interaction test in the open field, the social interaction time decreased in adolescent animals treated with LPS + MK-801&AMP. This promising result, suggesting the development of negative symptoms in predisposed individuals who had earlier experienced MK-801&AMP administrations, seems to be potentially consistent with the assumed relevance of the model to schizophrenia. Nevertheless, more research would be needed to draw a definitive conclusion. There was a statistically significant reduction of nose contact and crawling and a trend towards reduction of overall time spent in social interactions. In addition, we observed a decrease also in the remaining patterns of social behavior, including genital investigation, although not statistically significant. The genital investigation represents a natural propensity of rats to investigate and olfactorically discriminate conspecifics (Engelmann et al., 2011, Engelmann et al., 1995). Therefore, our findings suggest that the LPS + MK-801&AMP treatment may have decreased the motivation for social recognition in adolescent animals. Adolescence is a transition period of substantial neurobiological and behavioral changes. One specific psychological change is an intensification of emotional experiences. These heightened emotional experiences have been argued to be the basis of psychopathology (Casey et al., 2010). Therefore, it is not surprising that affective and psychotic disorders, including schizophrenia, arise during adolescence compared to other life stages (see (Brenhouse and Schwarz, 2016; Giedd, 2008; Owen et al., 2016)).

Conversely, we did not observe significant changes in social interactions in LPS + MK-801&AMP-treated adults despite their exposure to a higher number of MK-801&AMP administrations. In this age group, the results were not encouraging, raising questions about possible reasons. One possible explanation is that adolescence may represent a period of increased sensitivity to the employed pharmacological manipulations. The known increased sensitivity of adolescent rats to some behavioral effects of MK-801(Pešić et al., 2010) seems consistent with this notion. This result can also be related to the experimental approaches used in this study. Further exploration through additional tasks assessing social behavior could provide a more comprehensive understanding of the delayed impact of MK-801&AMP on the social behavior of adolescent and adult rats. Nevertheless, based on the current results, the social deficit in our model seems to be related to a specific ontogenetic period (adolescence) rather than to the higher number of MK-801&AMP administrations.

Furthermore, the study revealed an effect of age on social interaction. Adolescent control animals spent more time in overall social interactions than adults, aligning with the fact that social stimuli are more rewarding for adolescent than adult rats (Douglas et al., 2004). However, an exception was observed in the genital investigation, where adult rats spent significantly more time in anogenital investigation. Genital investigation time increases with age between PD32 and PD60 and remains the same after rats attain sexual maturity (Mikulecka et al., 2014).

The social impairment observed in LPS + MK-801&AMP-treated adolescents was detected only by the social interaction test in the open field, highlighting the importance of a careful selection of methodological approach. The varying results may be attributed to the distinct character and sensitivity of the tests used: in the social interaction test in the open field, the animals were allowed to interact closely, while in the social approach test in the Y maze, stimuli animals were confined, reducing the behavior of experimental animals primarily to approach/avoidance.

Numerous studies used different methodologies to evaluate the long-term effects of early-life immune activation on rodents' social behavior. Kirsten and colleagues (Kirsten et al., 2010) reported reduced play behavior in immature rats and decreased adult social interaction in rats exposed to LPS prenatally. Similarly, male rats prenatally exposed to LPS exhibited reduced time spent in social contact when tested at PD45 and PD90 (Vojtechova et al., 2021). Previous studies demonstrated that early-life LPS-induced inflammation at PD14 altered emotional behavior and decreased social behavior in PD40 adolescent rats (Doenni et al., 2016). In a previous experiment with the same neonatal LPS administration regime as in the present study, the treatment did not affect sociability assessed in 2-month-old animals in the social interaction test. However, contrary to our study, Vojtechova et al. (Vojtechova et al., 2018) did not use intact animals as stimuli rats in the social interaction test, and the age at assessment differed.

In the context of psychotropic drugs, MK-801 and some other N-methyl-D-aspartate receptor antagonists can model positive and negative symptoms of schizophrenia, including social withdrawal (Rung et al., 2005). However, the results are ambiguous due to variations in paradigms, doses, and designs (Gururajan et al., 2010, Jones et al., 2011, Wilson and Koenig, 2014). Acute or subchronic administration of MK-801 induced social withdrawal (Wilson and Koenig, 2014) and impaired sociability (Cieslik et al., 2019; de Moura Linck et al., 2008; Gururajan et al., 2010; Kamińska and Rogóz, 2015; Rung et al., 2005), although often at higher doses that might alter motor functions. Nonetheless, the studies primarily examined acute MK-801 administration, modeling psychotic-like episodes. The studies focusing on social behavior after withdrawal, potentially modeling the assumed long-term impact of psychotic-like episodes, which would be more relevant to our study, are scarce. Cieslik and colleagues reported decreased social interaction one day after completing the chronic (7-day) MK-801 dose regime (Cieslik et al., 2019). In contrast, Sams-Dodd failed to observe any changes in social interaction 7 days after chronic MK-801 administration (Sams-Dodd, 2004).

The psychostimulant AMP can model positive symptoms of schizophrenia in rodents, but its potential to induce social deficit is unclear (Gururajan et al., 2010, Jones et al., 2011, Rung et al., 2005). High doses of AMP led to decreased social interaction and increased aggression (Ellison et al., 1978, Gambill and Kornetsky, 1976). In addition, acute administration of AMP disrupted sociability (Šlamberová et al., 2015a) and social approach behavior (Hanks et al., 2013). On the other hand, other studies did not corroborate the disruption of social behavior after acute or chronic AMP administration (Rung et al., 2005, Sams-Dodd, 1998). However, less is known about the potential long-term consequences on social behavior persisting for days after a previous AMP application. In one such study, AMP did not affect social interactions during the withdrawal (Der-Avakian and Markou, 2010).

Regarding brain OT levels, we revealed an age-dependent variation in OT levels in the hypothalamus, with higher levels in adult animals. However, no significant treatment effect was detected, although there was a tendency to decrease in the adolescent LPS + MK-801&AMP group. The lower hypothalamic OT level could be possibly related to the reduced social interaction observed in this group. In this regard, interesting findings were described in the schizophrenia model induced by phencyclidine, sharing similar mechanisms of action with MK-801. Chronic phencyclidine administrations followed by 3-day withdrawal led to a social interaction deficit associated with decreased hypothalamic OT mRNA expression (Lee et al., 2005).

OT, a neurotransmitter of hypothalamic origin, targets limbic structures via axon collaterals, thereby regulating emotional behavior, social interaction, and cognition (Lee et al., 2009, Rich and Caldwell, 2015). Deficient maturation and dysregulation of the OT system due to adverse environmental factors in various life phases can contribute to the vulnerability to neurodevelopmental disorders, including schizophrenia (Johnson and Buisman-Pijlman, 2016, Rich and Caldwell, 2015). Moreover, lower OT levels have been associated with social withdrawal in schizophrenia (Johnson and Buisman-Pijlman, 2016, Rubin et al., 2010, Sasayama et al., 2012).

## 5 Conclusions

We have proposed a novel pharmacological two-hit model in male Wistar rats potentially relevant to schizophrenia and investigated its delayed effects on social behavior. The results showed that early immune activation (LPS) followed by two MK-801&AMP administrations, intended to mimic psychotic-like episodes, reduced adolescent social interactions. However, early immune activation followed by five MK-801&AMP administrations did not induce changes in social behavior in adult animals. This suggests that the pharmacological manipulations caused only transient age-dependent changes in social behavior. The model was thus, in certain aspects, promising, while other findings are less encouraging and require further investigation. Our findings highlight the critical importance of the methodological approach and selection of the ontogenetic period studied when investigating complex behaviors such as social behavior. Our results showed that animal models should include social behavior assays allowing animals to interact physically in a more naturalistic environment across multiple developmental time points. A combination of behavioral assays that aim to assess social communication (e.g. ultrasonic vocalization) and motivational and rewarding properties of social behavior in a more natural environment utilizing automated behavioral analysis technology could significantly contribute to advancing future research.

## CRediT authorship contribution statement

**Silvester Ponist:** Investigation. **Barbora Cechova:** Investigation. **Jana Jurcovicova:** Investigation. **Romana Slamberova:** Supervision. **Marketa Chvojkova:** Writing – review & editing, Investigation. **Kristina Holubova:** Writing – review & editing, Writing – original draft, Visualization, Formal analysis. **Anna Mikulecka:** Writing – review & editing, Writing – original draft. **Kristina Hakenova:** Writing – review & editing, Investigation. **Karel Vales:** Writing – review & editing, Supervision, Funding acquisition, Conceptualization. **Jiri Horacek:** Supervision.

## Compliance with ethical standards

The experiments were conducted following the guidelines of the European Union directive 2010/63/EU on the protection of animals used for scientific purposes, and were approved by the Animal Care and Use Committee of the National Institute of Mental Health, Klecany, Czech Republic (reference number MZDR 14764/2019–4/OVZ). All efforts were made to minimize the use of the animals and their suffering.

## Declaration of Competing Interest

The authors declare that they have no known competing financial interests or personal relationships that could have appeared to influence the work reported in this paper.
