# Supplementary material for: A new two-hit animal model for schizophrenia research: Consequences on social behavior
Source: IBRO Neurosci Rep. 2025 May 28;19:38–49. doi: 10.1016/j.ibneur.2025.05.012 (PMC12169724; doi:10.1016/j.ibneur.2025.05.012)
Supplement: Supplementary file 1 — Supplementary material [file mmc1.pdf]

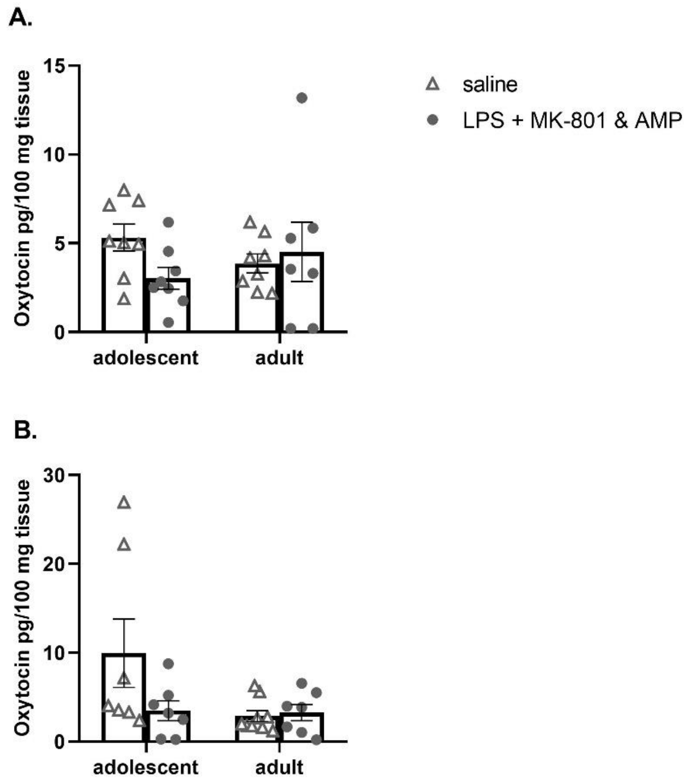

Suppl. 1: Oxytocin (OT) levels in the prefrontal cortex (A) and the striatum (B). No effect of the treatment, age, or their interaction on the OT levels was revealed. Mean + SEM values.
